# Retinoblastoma: present scenario and future challenges

**DOI:** 10.1186/s12964-023-01223-z

**Published:** 2023-09-04

**Authors:** Vishnu Vardhan Byroju, Aisha Shigna Nadukkandy, Marco Cordani, Lekha Dinesh Kumar

**Affiliations:** 1 Department of Biochemistry, American International Medical University, Gros Islet, St. Lucia, USA; 2 CSIR-Centre for Cellular and Molecular Biology, Habsiguda, Uppal Road, Hyderabad, India; 3 Department of Biochemistry and Molecular Biology, Complutense University of Madrid, and Instituto de Investigaciones Sanitarias San Carlos (IdISSC), Madrid, Spain

**Keywords:** Retinoblastoma, Knudson hypothesis, Chemotherapy, Cell plasticity, Signalling pathways, Molecular targeted therapies

## Abstract

With an average incidence of 1 in every 18,000 live births, retinoblastoma is a rare type of intraocular tumour found to affect patients during their early childhood. It is curable if diagnosed at earlier stages but can become life-threateningly malignant if not treated timely. With no racial or gender predisposition, or even environmental factors known to have been involved in the incidence of the disease, retinoblastoma is often considered a clinical success story in pediatric oncology. The survival rate in highly developed countries is higher than 95% and they have achieved this because of the advancement in the development of diagnostics and treatment techniques. This includes developing the already existing techniques like chemotherapy and embarking on new strategies like enucleation, thermotherapy, cryotherapy, etc. Early diagnosis, studies on the etiopathogenesis and genetics of the disease are the need of the hour for improving the survival rates. According to the Knudson hypothesis, also known as the two hit hypothesis, two hits on the retinoblastoma susceptibility (RB) gene is often considered as the initiating event in the development of the disease. Studies on the molecular basis of the disease have also led to deciphering the downstream events and thus in the discovery of biomarkers and related targeted therapies. Furthermore, improvements in molecular biology techniques enhanced the development of efficient methods for early diagnosis, genetic counseling, and prevention of the disease. In this review, we discuss the genetic and molecular features of retinoblastoma with a special emphasis on the mutation leading to the dysregulation of key signaling pathways involved in cell proliferation, DNA repair, and cellular plasticity. Also, we describe the classification, clinical and epidemiological relevance of the disease, with an emphasis on both the traditional and innovative treatments to tackle retinoblastoma.

Video Abstract

**Supplementary Information:**

The online version contains supplementary material available at 10.1186/s12964-023-01223-z.

## Introduction

With an ability to convert electromagnetic/ light energy to electrical energy, the retina acts as a transduction screen that enables the visualisation of any object in front of it by transmitting this electrical energy as nerve impulse to the cortical functioning centres [1]. It layers the innermost part of the eye and hosts various types of cells like rods and cones which are integral for its proper functioning. Various diseases like retinal tear, retinal detachment, diabetic retinopathy, macular degeneration, retinitis pigmentosa etc. have been associated with retina and its impaired functioning. One such disease is retinoblastoma, the most common malignant intraocular tumour in children. Retinoblastoma is theorized to arise from the cones of the retina, which have certain properties that leave them rather susceptible to tumorigenesis [2]. Globally, 1 in every 16,000 to 20,000 live births is known to be afflicted by retinoblastoma [3]. Most of the cases are diagnosed before the age of 5 and it accounts for 3% of all childhood cancers [4]. Of these, 200–300 new cases emerge in the United States alone and this incidence has not changed in over 40 years [5]. Previous studies indicated that there were significant differences in the incidence of retinoblastoma based on gender, ethnicity, and infections due to poor sanitation [6]. The newer studies, however, deny significance of such differences and consider retinoblastoma to have similar incidence throughout the world [4].

Despite being rare, retinoblastoma gained interest within the scientific community since RB1 gene is the first tumour suppressor gene to be discovered [7]. In its hereditary form, retinoblastoma is associated with de novo mutations resulting in tumours at other foci in the body which are termed ‘second primary tumours’ and is attributed to the role played by phosphorylated Rb protein [8]. Subjects with hereditary retinoblastomas are at a higher risk of developing other second primary tumours such as osteosarcomas, melanomas etc. [9]. Every form of retinoblastoma, familial and sporadic has the Rb gene mutated to some extent resulting in the downstream processing of aberrant transcripts [10, 11]. RB1 gene is located on the largest acrocentric chromosome, 13 and consists of 27 exons [9, 10]. Following transcription, Rb protein is formed and gets involved in the regulation of cell cycle at the G1-S checkpoint. Phosphorylation essentially acts to ‘switch off’ Rb tumour suppressor protein which results in [12] deregulation of downstream molecular events that eventually results in retinoblastoma [13].

Cell plasticity is a term usually referred to phenotypic and molecular changes resulting from genetic and epigenetic alterations responsible for cell variability and intra-tumor heterogeneity. Plasticity is a distinctive trait of tumor progression and represents an important evolutionary mechanism by which tumor cells can survive to environmental and energy stresses [14]. Concerning the functional significance, plasticity confers to cancer cells the ability to dynamically oscillate between different stages of differentiation and stemness with limited or high tumorigenic and metastatic potential [15]. Cancer cell plasticity is also linked to the epithelial-to-mesenchymal transition (EMT) program and metabolic rewiring from glycolytic metabolism to oxidative phosphorylation and involves a complex interplay between intracellular signaling pathways with environmental cues [16, 17]. The major signaling pathways involved in retinoblastoma initiation and development are Rb, p53, Ras/MAPK and Notch pathways [18, 19]. These belong to an oncogenic network that promotes phenotypic and molecular changes in cellular state to adapt retinoblastoma to extracellular stress and drug treatment. As described below, such pathways may lead to EMT activation [20], undifferentiated stem-like cellular state [21], or glycolytic shift towards OXPHOS to alternate energy fuels and metabolic routes depending on the need of tumor cells [22, 23]. The deep understanding of the mechanism underlying cell plasticity in retinoblastoma may provide insights for establishing new therapeutic interventions.

Like many other diseases, economic disparities find their way into dictating presentation and survival rates in cases of retinoblastoma too across the world. Age group of subjects that present with signs of retinoblastoma is higher in middle and low-income countries as compared to affluent nations, suggesting deficit in diagnostic resources [24]. Survival rates also portray a similar picture with developed nations nearing 90%, middle income countries averaging 70% and low-income countries about 40%. (Income disparities were based on the World Bank classification) [25, 26]. Financial constraints act as a major deterrent contributing to the delay in diagnosis and treatment in developing countries. Social stigma in certain cases also played a role in refusal of enucleation and resulted in the cancer metastasis. Availability of a cancer registry, infrastructure, multidisciplinary approach, medications, treatment noncompliance are known to be some of the major determinants of survival disparities [26].

## Classification of retinoblastoma (WHO classification)

**Fig. 1:**
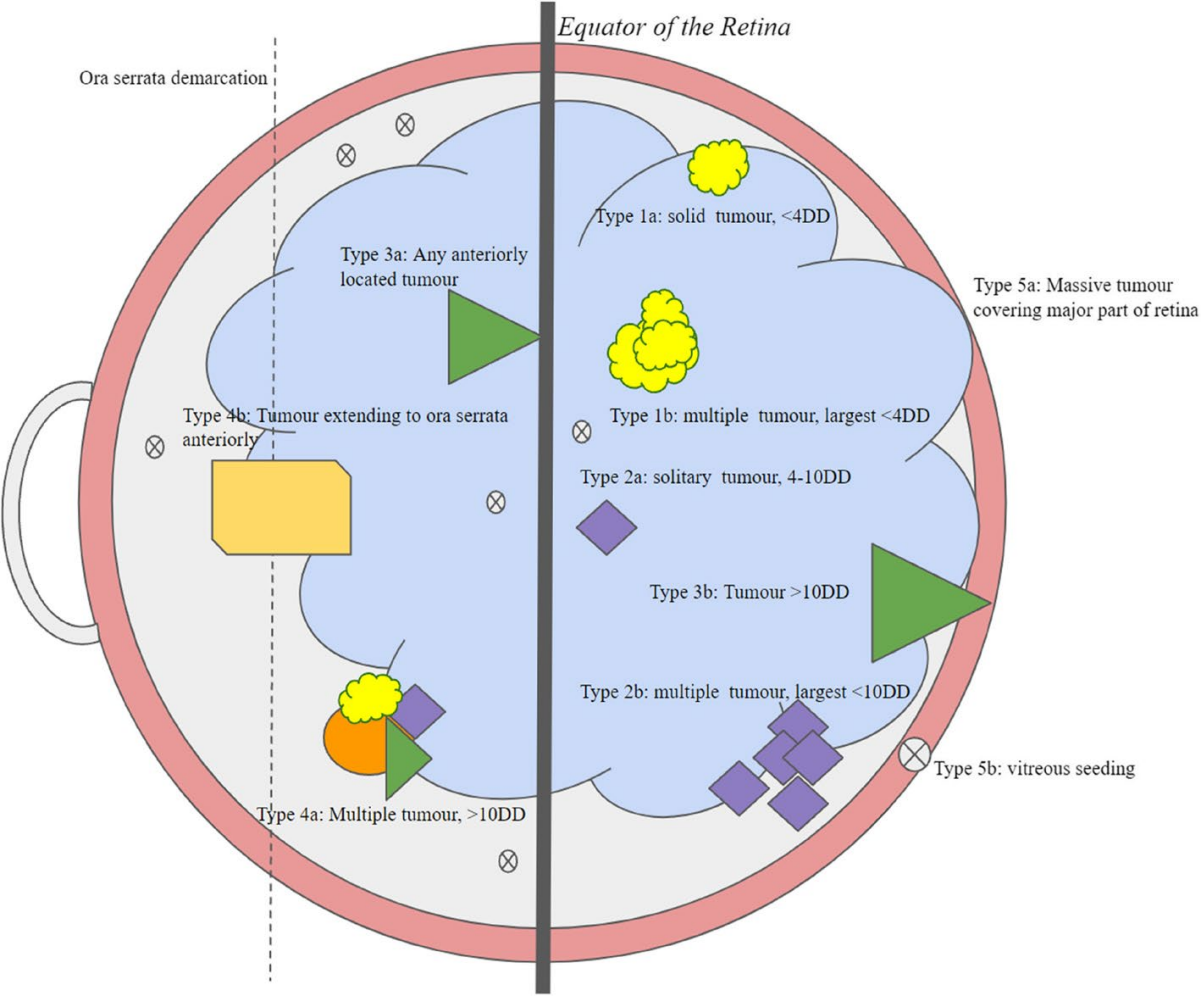
Pictorial representation for Reese Ellsworth’s Classification of Retinoblastoma. A graphical abstract of the Reese Ellsworth’s classification system prepared using arbitrarily chosen symbols to represent their corresponding groups within the classification system and to understand different demarcations of the eye. The 1a (yellow cloud shape) aims to represent a tumour behind the equator, solitary and less than 4-disc diameters in size; 1b (multiple yellow cloud shapes) aims to represent multiple tumours behind the equator of the retina with the largest tumour not exceeding 4-disc diameters.; 2a (purple diagonal shape) aims to represent a 4-10DD tumour behind the retina; 2b (Multiple purple diagonal shapes) represents multiple tumours between 4-10DD; 3a (Green triangle shape) aims to represent a tumour anterior to the equator of the retina; 3b (Green triangle shape in a different location) aims to represent a > 10DD tumour behind the equator; 4a (multiple shapes of varied colours) aims to represent multiple tumours with some of them larger than ten-disc diameters in size; 4b (yellow slip diagonal shape) aims to represent a tumour that is crossing the ora serrata; 5a (Large, undefined blue shape in the background) aims to represent a large tumour that covers more than half of the retina; 5b (X marked at the border of the figure and in multiple areas marked across the eye figure) aims to represent vitreous seeding of the eye

Retinoblastoma had major classification overhauls and has finally stabilized with the Reese-Ellsworth and ICRB (International Classification of Retinoblastoma) as the most accepted form as of now. Reese-Ellsworth (R-E) was considered to be the first classification done for intraocular cancer and was developed to predict the chances of saving the eye following external beam radiotherapy (Fig. 1; Table 1). However, in the 1990’s, when intravenous chemotherapy for intraocular retinoblastoma was introduced, the R-E classification system was no longer appropriate. Therefore, a new classification scheme referred as “International Intraocular Retinoblastoma Classification” (IIRC) was developed [27]. The IIRC scheme, groups the tumours into A to E classes depending on their size, location, and other features like the presence of small colonies of cancerous cells in the vitreous (retinoblastoma seeds) and the presence of retinal detachment. This staging was further modified to form the Intraocular Classification of Retinoblastoma (ICRB), which differed from the IIRC mainly in the definitions of the advanced groups. Even though well accepted, certain discrepancies have been reported with ICRB affecting 5.2% of the group under study [28]. While RE and ICRB are 5 stage classification systems, the ICRB provides a better distinction between the severely affected eyes and the eyes that can be salvaged with less effort (Table 2).

**Table 1:** Tabular representation of the Reese Ellsworth’s Classification of Retinoblastoma

**Groups**	**Likelihood of salvage**	**Features used to classify the tumour**
I	Highly favourable	1a: Solid, solitary tumour that is less than 4-disc diameter in size and is present behind the equator or at the equator.
		1b: Multiple tumours with the largest of them not exceeding 4-disc diameters in size present behind or at the equator.
II	Favourable	2a: Solitary tumour behind or at the equator ranging between 4–10-disc diameters in size.
		2b: Multiple tumours ranging between 4–10-disc diameters in size behind the equator.
III	Doubtful	3a: Tumour is anterior to the equator of the eye.
		3b: Single tumour that is larger than 10-disc diameters behind the equator.
IV	Unfavourable	4a: Multiple tumours with some being larger than 4-disc-diameters in size.
		4b: Tumour extending past the ora serrata of the eye
V	Highly unfavourable	5a: Tumour large enough to involve more than half of the retina.
		5b: Vitreous seeding

**Table 2:** International Classification of Retinoblastoma and Prediction of outcomes of surgery

**Groups**	**Criterion**	**Number of Patients in group based on the study **[49] ***n* = 249**	**Treatment and success rate**
Group A	Retinoblastoma tumor with size < = 3 mm.	23 (9%)	Chemo reduction with vincristine, etoposide and carboplatin for 6 cycles (CRD) plus thermotherapy or cryotherapy. 100%
Group B	RB tumor with size > 3 mm, has a location close to the macula or presents with small amount of subretinal fluid.	96 (39%)	CRD plus thermotherapy or cryotherapy. 93%
Group C	Eyes with localised seeding of the tumor.	21 (8%)	CRD plus thermotherapy or cryotherapy. 90%
Group D	Diffuse seeding of the tumor within the eyes.	109 (44%)	CRD plus thermotherapy or cryotherapy. 47%
Group E	Huge mass of the tumor covering a great proportion of the eye.	0 as the patients needed management with enucleation and hence were excluded from the study.	Enucleation with CRD in certain cases.

Furthermore, retinoblastoma has been assigned various stages depending upon the progression of the disease and its potential for metastasis. The system was named International Retinoblastoma Staging System (Table 3) where, the stage 0 often shows good prognosis with treatment while stage IV shows poor outcome. During stage IV, the cancer is considered to be extraocular and can lead to bulging out of the eye [29]. When it comes to differentiating it into various types, the disease can appear as unilateral, bilateral or trilateral. The chance of a patient developing a trilateral retinoblastoma is 6% higher in case of bilateral retinoblastoma as compared to unilateral and can be fatal in 50% of the cases. Another classification of retinoblastoma could be based on direction of progression of the disease; i.e. exophytic if the tumour originates in the retina and spreads in the direction of the brain behind or endophytic if the tissue spreads in an anatomic anterior direction.

**Table 3:** International Retinoblastoma Staging System (IRSS) [97]

**Stages**	**Clinical description**
0	Conservative treatment of patient
I	Enucleated eye, complete resection histologically
II	Enucleated eye, microscopic residual tumour
IIIa	Extends regionally, overt orbital disease
IIIb	Extends regionally, pre-auricular or cervical lymph node extension
Iva	Metastatic haematogenous
	1. single lesion
	2. multiple lesion
IVb	Extends to CNS
	1. pre-chiasmatic lesion
	2. CNS mass
	3. leptomeningeal and cerebrospinal fluid disease

## Epidemiology

Most of the estimated incidence of retinoblastoma varies by country from 3.4 to 42.6 cases per million live births. In the United States alone, the incidence is 11.8 affected per million live births among children less than 5 years of age. This accounts to a global average of 8200 new cases per year, out of which 60% of cases are unilateral and 40% are bilateral. The unilateral retinoblastoma (most of which are sporadic) is diagnosed at a median age of 2 years while bilateral one is diagnosed earlier, at nearly 1 year of age [3]. Retinoblastoma has an autosomal dominance pattern with each offspring having a 50% risk of inheriting the RB1 gene mutation that has a 90% penetrance. Family history is positive in only 10% of the individuals, both in case of unilateral and bilateral retinoblastoma and these individuals are considered to have the heritable form. If an individual has heritable retinoblastoma, his or her siblings should be tested for germline mutation, presence or absence of which determines the future risk. If no mutation is found, the risk is very similar to the general population and if a mutation is found, the high risk necessitates periodic surveillance [30].

The future of survivors of retinoblastoma is tough as they have chances of developing other cancers due to metastasis or germline mutation. Even though there is survival probability of 9 out of 10, most of them turn blind. A stable incidence rate has been observed in the United States for about 40 years from 1973-to 2012 based on a report [31]. The incidence of retinoblastoma has been increasing in European nations when compared to previous reports. A multinational European study postulates that such an increase is linked to improved survival of familial subjects owing to advancement in the health care sector and the availability of resources [32]. A retrospective cohort study using the Finnish Cancer Registry (1964–2014) showed an increase in familial retinoblastoma cases while the overall incidence remained the same [3]. Canadian reports represents a stable incidence of retinoblastoma which is comparable to that of other developed nations [4]. Poland demonstrates a similar pattern of incidence and management of retinoblastoma to that of western Europe and North America despite being middle-income nations [5]. The lack of availability of registries has limited our understanding of trends in Africa. A higher incidence of retinoblastoma has been found in white population of South Africa, probably due to better access to diagnosis and management [6]. Studies from Southeast Asia were insufficient and demonstrated socioeconomic barriers for their access to health care system. Poor awareness, lack of training for health care workers to detect retinoblastoma early, and comorbidities resulted in late diagnosis and complex management [33]. A study of the incidence of retinoblastoma in Taiwan demonstrated no notable trend from 1998–2011. An initial declining trend from 2005 to 2011 followed by an increase in the incidence of retinoblastoma was noted in Lebanon. An increase in awareness among health care professionals and taking refugee data into consideration are some essential steps for further understanding of disease epidemiology [26]. Retinoblastoma presenting at later stages is common in developing countries and examination of children during primary care visits is essential to improve diagnosis and survival [25].

## Screening and diagnosis of retinoblastoma

**Fig. 2:**
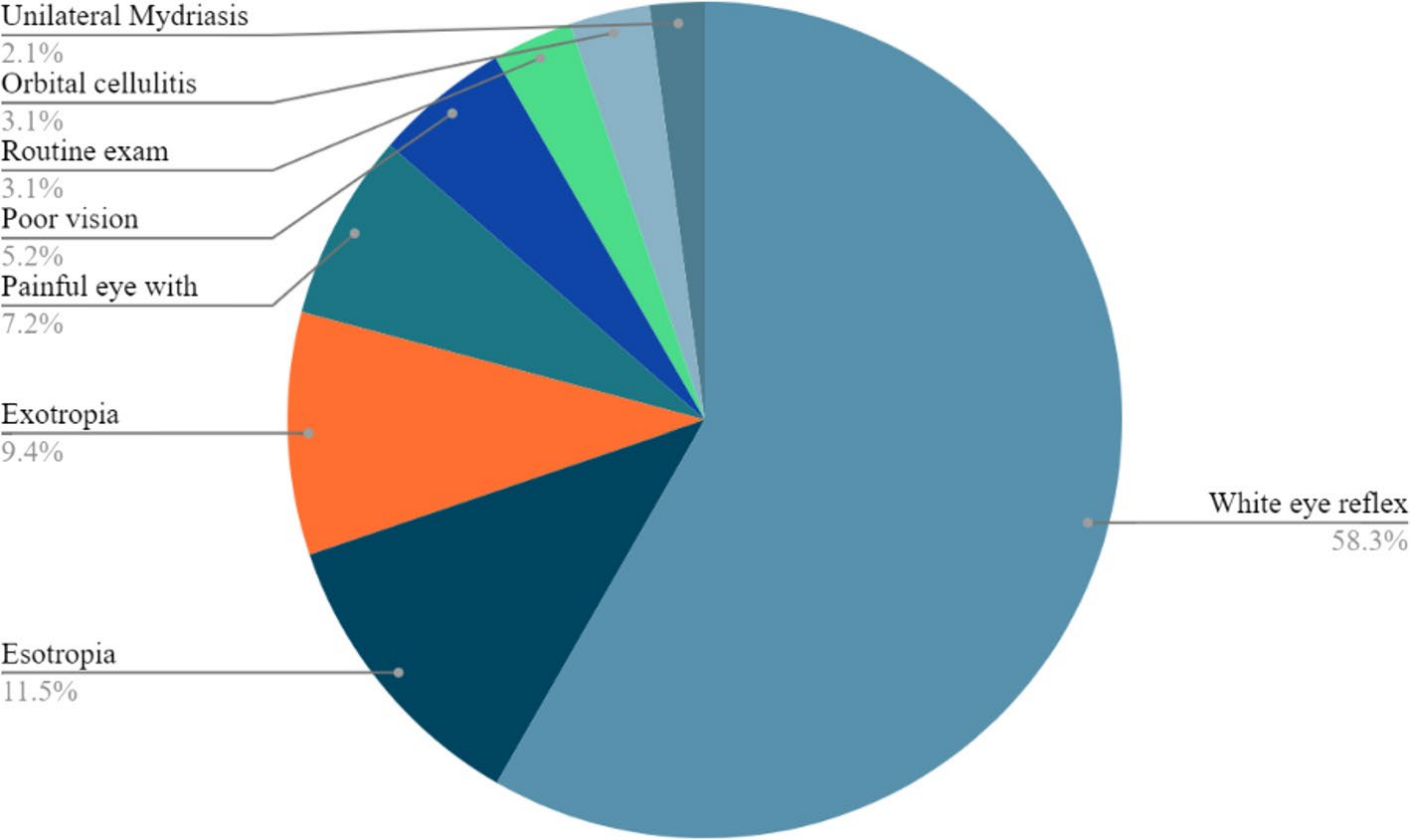
Symptoms of retinoblastoma. Pie chart representation of the presenting symptoms of retinoblastoma

Screening of retinoblastoma involves various tests for detecting the symptoms (Fig. 2) of retinoblastoma. Leukocoria (sometimes referred to as the ‘cat’s eye reflex’) can be detected by the presence of a white reflection in photographs or the red reflex test. A simpler approach would be to use mobile phones for the same purpose where, such an application exists in the form of an app called “White eye Detector” developed by Bryan Shaw. Performing cover test detects the presence of strabismus while all other signs can be identified by a systematic visual examination. Screening by an ophthalmologist is a necessity in children with a positive family history of retinoblastoma. Offspring and siblings of affected patients require regular screening examinations in childhood unless genetic testing is done to rule out a gene mutation in which case the risk is similar to that of the general population. Genetic counselling for families with retinoblastoma can help determine the risk to future offspring and whether other family members are at risk of developing the disease [34].

An ophthalmoscope view shows the optic disk, the physiological cup, the retinal vessels, the macula and the fovea centralis so the tumour is quite easily identified. Non-invasive two dimensional and three-dimensional real-time ultrasound techniques have eased detection significantly. Once the polymerase chain reaction (PCR) was invented, it became easy to identify the batch deletion of exons in chromosome no. 13 of the children whose parents carry the RB1 gene mutation. Quantitative multiplexing is an advanced form of the technique where several primers are used at the same time together to identify batch deletions [35]. Use of X Ray and Computed Tomography has found its place in detection of retinoblastoma relying on properties such as calcification in the latter technique. Gadolinium contrast enhancement followed by MRI scan has been helpful to detect the tumour. Fat saturation was quite helpful as well wherein the fat was suppressed at one time and was expressed at another time in anticipation of finding the nature of the tumour. T1 weighted imaging with a magnetization of 63% was used for better results [36, 37].

Fluorescein Angiography is a technique that requires injection of a small bolus of fluorescent material into the blood stream which finally reaches the ophthalmic artery. A specialized camera takes pictures of the eye once it reaches there through the catheter using the fluorescence of the arteries of the eye as a light source [38, 39]. Unsuccessful attempts at detecting retinoblastoma using positron emission tomography (which relied on detecting increased glucose consumption by the tumour) were made using Flourine-18-fluorodeoxyglucose throughout many years. The conclusion that FDG-PET is not currently widely established and does not provide any significant advantage over MRI/CT except in metastasis rendered MRI as the gold standard [40]. Early detection using an ultrasonogram in-utero to study the face and eyes can help in detection of the cancer, resulting in earlier detection and potential cure [41].

## Treatment and management of the disease

Retinoblastoma is a cancer that can be cured if diagnosed at an appropriate time. The involvement of structures beyond the retina and the vitreous humour should be taken into consideration as they have the potential to progress into metastasis rapidly. The treatment of retinoblastoma is often complex and involves decisions to be made based on a number of factors including but not limited to the size of the tumour in various axes, age of the patient, risk of secondary metastasis, previous attempts made at chemotherapy, toxicity of the chemotherapeutic agent in the subject and laterality of the tumour [42, 43].

### Enucleation

Complete removal of the eye that is affected by the tumour, or surgical excision is termed as enucleation. It is the least conservative possible management and hence reserved for cases that cannot be helped otherwise. Enucleation should be avoided in cases where salvage is possible with other treatment modalities in an effort to preserve vision and improve conservation. For the first 2 years after surgery, all patients undergoing enucleation must be carefully monitored for the risk of orbital relapse. Hydroxyapatite implants coated with specialized polymers and have attachment sites for the extraocular muscles are being implemented. Overall, enucleation was widely used and continues to be used in cases where other treatment modalities are unhelpful [44].

### Intra-arterial (IAC) chemotherapy

Pierre Gobin et al. have reported their overall experience with intra-arterial chemotherapy to be safe and effective in avoiding enucleation [45]. Modern microcatheter techniques were used to administer the chemotherapy agents and success was obtained with acceptable levels of toxicity. The choice of the drugs included mephalan, topotecan hydrochloride, carboplatin and methotrexate in this route of administration. The Kaplan Meier curves estimated the event free survival of receivers of the IAC route to be higher than other treatment modalities. Since deleterious after-effects of IVC were observed, IAC could successfully replace IVC route and furthermore it delivered a higher dose of chemo agents to the target site [45]. Intra-arterial chemotherapy is administered as either primary or secondary treatment interventions and studies reveal no significant difference in post-treatment vascular events. Since post chemotherapeutic toxicity was observed, there is a need for careful surveillance. Although intra-arterial chemotherapy is proving to be a strong treatment modality, its limitations require the implementation of better techniques [5]. IAC efficacy is decreased in cases of extensive collateral meningeal vascular presence due to dilution of the agent into these vessels. Collateral blood supply to the retina apart from the ophthalmic artery has variations and has implications in the dose delivery. Catheterization of relevant arteries requires skill and the technical difficulties could arise that can reduce the delivery of intra-arterial chemotherapy [46].

### Intravitreal chemotherapy (IVitC)

Focused drug delivery to a vitreal seed hotspot is considered precision intra-vitreal chemotherapy and is an emerging technique [32]. Often used as an adjunct therapy to IAC, in IVitC, drugs are delivered directly into the vitreous cavity in advanced stages of retinoblastoma where vitreous seeding occurs. A combination of chemodrugs such as melphalan and topotecan [33] are the preferred mode of treatment when tumour seeds recur in the vitreous and is found to be more effective than the respective individual treatments [42, 43]. While the presence of vitreous seeds is an indication for Intra-vitreal chemotherapy, contraindications include diffuse dispersion of the tumour seeds, invasion of the anterior chamber of the eye, hemorrhage into the vitreous humour, and secondary glaucoma [6]. Acute hemorrhagic retinopathy and heterochromia, the presence of different iris colours has been noted secondary to intra-vitreal melphalan and topotecan administration [25, 26]. With the advent of nanoparticle delivery, intra-vitreal chemotherapy has promising prospects in the management of retinoblastoma [46].

### Thermotherapy

This mode of treatment is often engaged for tumours of minor dimensions, not exclusively for the eye. The usual dimension as indicated for thermotherapy alone is of a diameter of maximum 4 mm. Diode system delivers infra-red rays either through the pupil or the sclera to destroy tumour tissue by applying focused heat to induce necrosis. Ideal temperature of thermotherapy ranges from 45–60 ^C^. It is often used in conjunction with other treatment modalities such as chemotherapy [47]. Complications are seen in some cases which include but are not limited to atrophy of the iris, obstruction of the retinal vein and detachment of the retina. Attempts were made to avoid thermotherapy as a single modality in cases where seeding of the vitreous humour is observed. Lasting regression is seen in 86% of the subjects in selected cases [48].

### Cryotherapy

Much like thermotherapy, cryotherapy is an adjuvant and used in conjunction with other treatments. A retinoblastoma tumour up to 3.5 mm in diameter and 2 mm in thickness could be treated with cryotherapy. This therapy is contraindicated in cases with vitreous seeding and any tumour that has dimensions larger than the norm. The modality involves the application of triple freeze thaw technique using liquid nitrogen [49].

### External beam radiation (EBR)

External beam radiation is in the line of management of treatment for retinoblastoma after enucleation as an attempt to salvage the remaining eye. A high energy photon beam or electrons are delivered at an angle where the tumour has maximum exposure. Complications include cataracts, conjunctivitis, dry eyes and intractable glaucoma. Considering side effects such as new mutations, dry eyes, keratopathy, retinopathy and optic neuropathy, EBR therapy is better restricted to extra ocular tumour extension or if better alternatives are available, this could be completely avoided [50]. Tumours can sometimes arise secondarily due to radiation exposure, however, development of new modalities of beam radiation reduce such instances [51].

Tumour regression should be followed up closely and the appearance, size, location, and number of tumours documented during each examination have to be assessed. When a tumour regresses after treatment, it can either appear as a white coloured calcific mass or as translucent piece of flesh. In most of the cases, patients undergo examination under anaesthesia every 4 to 8 weeks until the age of 3, followed by less frequent examinations if the disease is found to be latent. Recurrence of the disease has been found to occur years after treatment and is very common these days. Patients with hereditary retinoblastoma also require long-term follow-up because such patients have an increased lifetime risk of developing secondary malignancies [52].

## Retinoblastoma: the genetics behind the disease

### Ascertaining the ‘two-hit’ hypothesis

Retinoblastoma gene is best known as the tumour suppressor that inspired the ‘two-hit’ hypothesis. The idea that the loss of both the alleles of a tumour suppressor gene is a pre-requisite for tumour initiation occurred to Knudson in the 1970s. Fifteen years later in 1986, retinoblastoma gene (RB1) was localized and cloned for the first time, confirming Knudson’s prediction [7]. Located on chromosome 13q14, spanning over a length of 190kbp, RB1 is the most important carrier of mutations in the malignant tumour affecting the retina of mostly young children. Present in two distinct clinical forms, retinoblastoma is a genetic disease that is either inherited or occurs sporadically. In hereditary retinoblastoma, an inherited germline mutation in the patient is followed by an acquired mutation in the developing retinal cells, leading to the completion of the ‘two hits’. The sporadic version of the disease is followed by separate mutations on both the alleles in the retinoblastoma gene of the somatic cells [53]. It was observed that patients with hereditary form of the disease often have their both eyes affected by multiple tumours while in non-hereditary form, patients get affected unilaterally. The unilateral tumours are formed due to the double mutations occurring as a result of the somatic events in the retinal cells after fertilization. This becomes unifocal if only a single retinal cell is affected while, if the mutation occurs during the process of development and in turn multiple cells are affected, the retinoblastoma appears multifocal. But then, not all unilateral tumours are formed as a result of somatic events. Studies suggest that around 15% of the unilateral retinoblastomas are formed in patients with a hereditary mutation in one of the alleles and a constitutive one in the other [54]. In case of bilateral retinoblastoma, the RB1 gene, in most of the patients, acquires a de novo mutation during spermatogenesis, indicating that it is mostly the paternal chromosome that is vulnerable to mutations [55]. Furthermore, studies on both hereditary and sporadic retinoblastoma have suggested that the polymorphic markers flanking either sides of the RB1 gene often undergo loss of heterozygosity as a result of the second hit [56].

### The broad spectrum of mutations with respect to retinoblastoma

More than 50% of the mutations found on RB1 have been detected only once, indicating the broad spectrum of RB1 mutations that spread across promoter, most exons, and splicing regions of introns [57, 58]. Even though wide varieties of genetic variations like chromosomal rearrangements, large exonic deletions, hypermethylation of the gene promoter region, small length mutations, and single nucleotide substitutions occur in RB1, the majority of familial RBs are formed due to nonsense and frameshift mutations resulting in a loss of function. The premature halting of the translation leads to unstable mRNA mutants, further leading to no detection of functional retinoblastoma protein. Furthermore, comparative genome hybridisation and karyotypic analysis have depicted the loss as well as the gain of regions in the RB gene. Some cancers show a loss at 16q22 position, and others a gain of the region at 1q31, 6p22, 2p24-25, and 13q32-34. While 60% of retinoblastomas show a gain at 6p, a loss of 16q is found in children diagnosed at an older age. Studies on the gain or loss of regions have hastened the process of identifying various oncogenes and tumour suppressors involved in retinoblastoma initiation and progression [59].

The role of mosaicism is widely getting recognised in retinoblastoma as the genetic analysis of the disease is becoming more advanced [62]. Mosaicism is that genetic condition where the multicellular organism has more than one genetic line as a result of mutation. Mosaic retinoblastoma mostly occurs as a result of DNA damage during cell division. The adversity of the disease depends on the number of cells that are affected by the mosaicism. Usually the chance of the unilateral proband to have heritable retinoblastoma is precluded by testing for two mutant alleles in RB tumour DNA and then confirming the presence of these mutant alleles in their blood DNA. The absence of RB1 mutant allele in the blood of a parent with an affected child is where mosaicism acts as a causative reason. The chances of this becoming heritable are less as the mosaicism should occur in the embryonic stage [58].

Most often, the gene inactivation on RB1 is insufficient for the development of the disease. Studies over the past years show that additional genetic and stochastic events leading to the proliferation of retinal precursor cells are necessary to initiate tumorigenesis [60]. Though thousands of RB1 mutations have been documented, a small fraction of the unilateral retinoblastomas are found to have mutations that are yet to be identified in RB1 gene, indicating that there also exists an alternative mechanism for retinoblastoma genesis. Studies done in the early 2010s showed that these tumours formed in the retina, lacked RB1 mutations and instead showed amplification of MYCN gene. This is just considered to be an initiating event, and studies are still going on the development of MYCN amplified tumours [58]. Other genes that are inactivated are the leukemic oncogene DEK, Cadherin-11, and the transcriptional factor E2F3 [61]. With a two-fold increased expression, the kinesin gene KIF14 is overexpressed mainly in patients diagnosed at an older age. KIF14 and E2F3 activation is triggered due to the genomic instability caused by the RB1 mutation in many cases [62]. The senescence protein p16INK4A, involved in RB progression, is found to be absent in cells that escape from senescence. Similar research on the subsidiary mechanisms involved in the development of the disease through various gene expression studies has pointed out the necessity to decipher the complexity of the disease [63]. Hence there is an immense need to analyse various signalling pathways that are deregulated as a result of the disease, or vice versa.

## Signalling pathways involved in Retinoblastoma

**Fig. 3:**
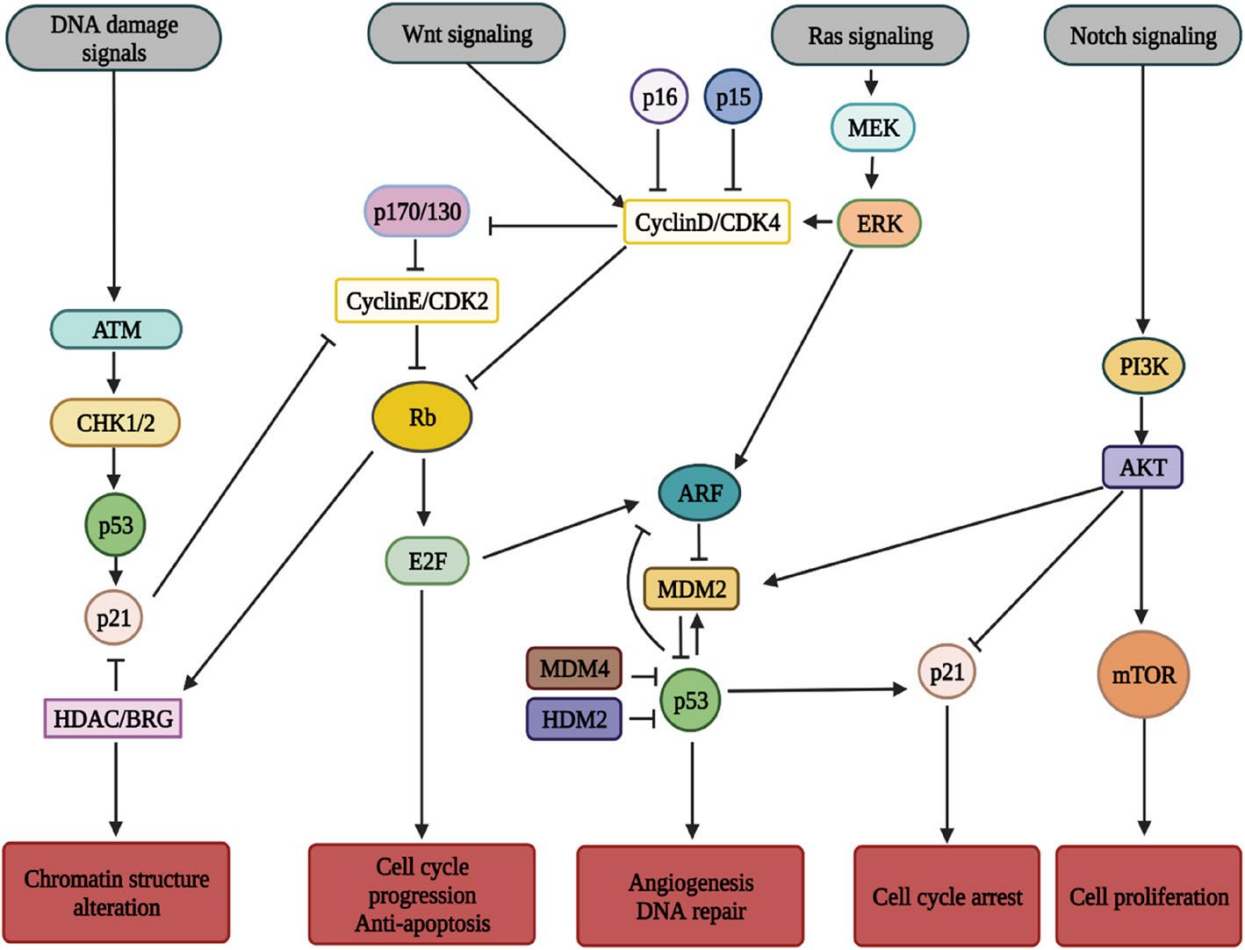
Retinoblastoma cancer signalling. Major signalling pathways that undergo concomitant deregulation during retinoblastoma (Created using Biorender) The abbreviations ATM (Ataxia Telangiectasia Mutated), CHK1/2 (Checkpoint Kinase 1/2), HDAC(Histone Deacetylase), BRG (ATP-dependent chromatin remodeler SMARCA4), CDK2(Cyclin Dependent Kinase 2), MEK(Mitogen-activated protein kinase kinase), ERK (Extracellular signal-regulated kinase), ARF (Alternate open reading frame), MDM2 (Murine Double Minute 2), MDM4(Murine Double Minute 4), HDM2(Human Double Minute 2), AKT (Protein kinase B), mTOR(Mammalian Target of Rapamycin)

Initiation of any tumour requires a set of genetic aberrations that regulate their basic cellular functions. These genetic and epigenetic changes allow the cells to escape their homeostatic controls further allowing them to proliferate outside their normal niche. This is mainly due to the concomitant dysregulation of various signal transduction pathways like, Rb, p53, Wnt, Ras-ERK, etc. (Fig. 3) which may have an impact on the oncogenic properties of retinoblastoma through multiple mechanisms, including the acquisition of stem cell-like phenotype, EMT activation and metabolic rewiring [20–23].

### Rb pathway

The transcription of 190kbp RB1 gene leads to the formation of a 110 kDa nuclear phosphoprotein renowned for its role in cell cycle regulation. It suppresses cell division by targeting the E2F family of transcription factors [64]. When hypo-phosphorylated, Rb binds to E2F and it prevents the transcription of genes necessary for G1 to S phase transition. When in need of cellular replication, the activated cyclin-cyclin dependent kinase (CDK) complex phosphorylates Rb and prevents its interaction with E2F. Mutations on the RB1 gene inhibit the Rb function, thus leading to the activation of E2F mediated transcription and hence, constant cell division leading to the retinoblastoma. Rb also binds to chromatin remodelling proteins like histone deacetylase (HDAC) and protein brahma homolog 1 (BRG) in its dephosphorylated form, causing alteration in chromatin structure and preventing access to transcription sites essential for cell cycle progression [64].

Studies have shown that inactivation of RB1 enables constitutive expression of E2F protein, causing a halt in the cell cycle control [65]. A recent study on murine models found that E2F independent CDK2 inhibition is a critical function required for p107 mediated tumour suppression. Inactivating this CDK2 inhibitor or deleting p27 expression was thus found to induce retinoblastoma. CDK2 being a protein known to cause tumour penetrance in all retinoblastoma models, removal of p107 in an Rb knockout model causing activation of CDK2 and post transcriptional induction of S-phase kinase-associated protein 2 (SKP2) further established the crosstalk among these proteins [66].

**Fig. 4:**
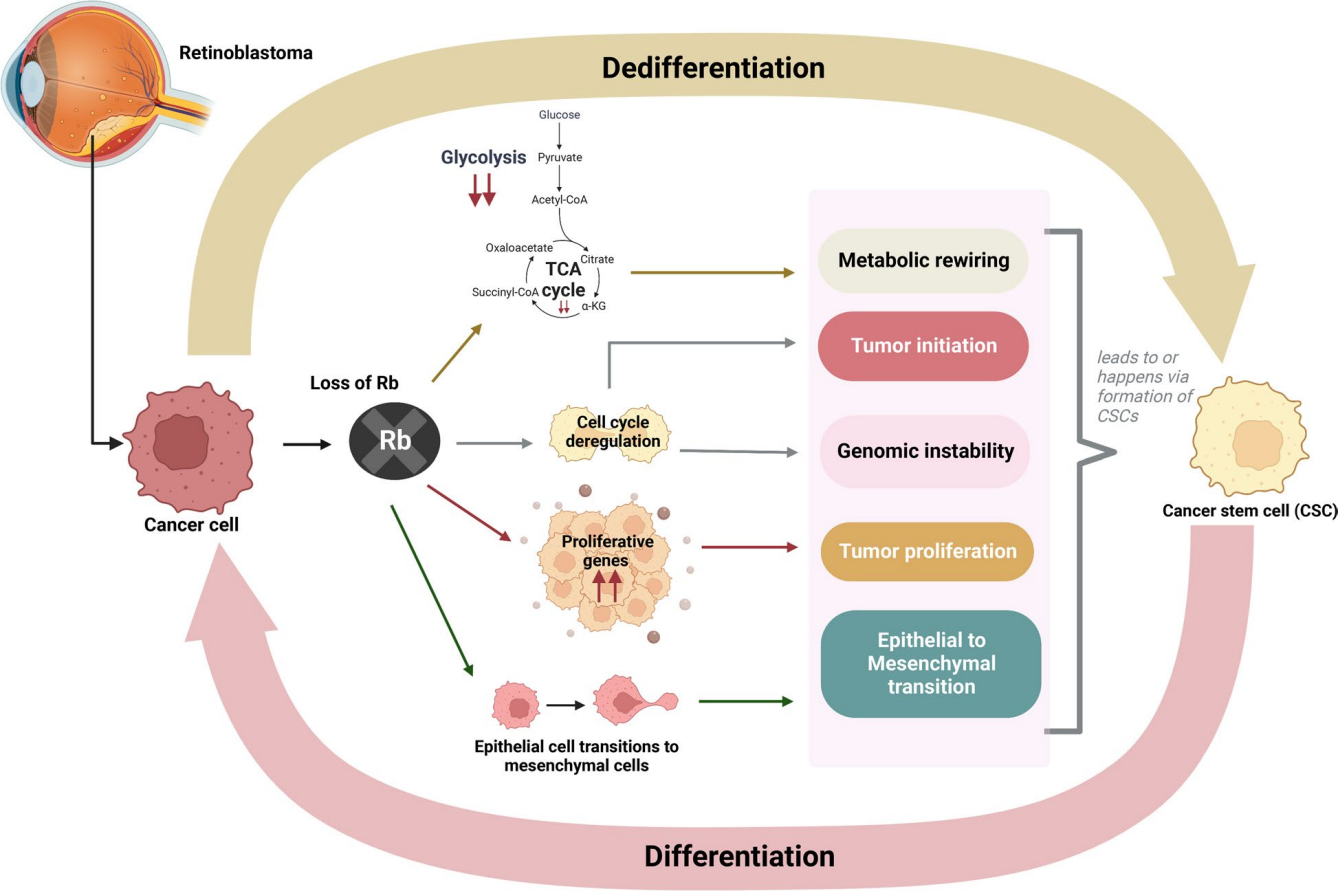
Loss of Rb gene in retinoblastoma. Graphical abstract on how loss of Rb gene can deregulate metabolic pathways, cell cycle and epithelial to mesenchymal transition (EMT) leading to formation of cancer stem cell and in turn enabling the development of the tumor. (Created using Biorender)

A number of studies linked dysregulation of Rb signaling pathway to the acquisition of multiple cellular status, as an undifferentiated “stem-like” phenotype, the activation of EMT program, or metabolic rewiring (Fig. 4). This highly dynamic phenotype might contribute to the oncogenic properties of retinoblastoma and lead to therapy resistance and inefficacy. For example, genetic, or functional inactivation of Rb family proteins in tissue stem/progenitor cells, impacts differentiation, sustains self-renewing capabilities thus promoting stem cells expansion and cancer initiation [67]. On the other hand, loss of Rb in differentiated post-mitotic cells has been observed leading to cell cycle re-entry and dedifferentiation, as well as to tumour initiation. In this regard, both mouse and human fibroblasts with inactivated Rb proteins acquire stem cells-like features, including the ability to form spheres and express inducible pluripotent genes [68, 69]. Interestingly, the Rb-E2F1 complex has been reported to counteract the expression of SOX2 and other stem cell-related molecules through the binding of their promoters and regulatory sequences [70]. Overall, these studies suggested that Rb inactivation not only exclusively leads to tumour formation by cell cycle deregulation but also by favoring the acquisition of self-renewal and stemness properties.

Epithelial Mesenchymal transition (EMT) leads to the acquisition of mesenchymal phenotype from epithelial cells and may occur during embryonic development, tissue regeneration or in cancer progression. EMT endows cancer cells of invasive and metastatic potential through highly regulated molecular pathways in which microRNAs, epigenetic and posttranslational regulators participate [71]. The impact of Rb family proteins in EMT is controversial. For example, Rb depletion in breast cancer cells limit cell-cell adhesion and induces a mesenchymal-like phenotype. In contrast, ectopic expression of Rb resulted in increased E-cadherin, which is required in epithelial cell-cell adhesion [72]. On the other side, Egger et al. found that Rb dephosphorylation in 3D cultures of invasive fibrosarcoma cells counteracts EMT, suggesting that targeting Rb phosphorylation in mesenchymal cancer cells might attenuate the invasiveness of cancer cells [73]. Recently, lack of Rb proteins in retinoblastoma tumours and in oncogene-induced senescent cells has been linked to a reduction of glycolytic genes and metabolic rewiring towards glycolysis independent energy production and mitochondrial activity [23]. On the other hand, loss of Rb1 may also enhance glycolytic metabolism in *Kras*-driven lung tumours [74]. Although the role of Rb proteins in metabolism is controversial, their loss may contribute to different metabolic status to alternate fuels and energy production mechanisms.

### p53 pathway

The p53 signalling pathway responds to various intrinsic and extrinsic stress signals by monitoring DNA replication, chromosome segregation and cell division [75]. Studies conducted over the years show that retinoblastoma caused due to RB1 mutations usually bypass the p53 pathway as they are already death resistant [63]. The activation of E2F in the absence of Rb leads to the overexpression of p14^ARF^. The p14^ARF^ protein further leads to activation of MDM2, an inhibitor of p53. The proteosomal targeting of Rb being a common neoplastic strategy often put forward, the ability of MDM2 to mediate the interaction of the proteasome with Rb enables proteasome-dependent ubiquitin-independent degradation of Rb that is taken into advantage [19]. The knocking down of MDM2 results in hypo-phosphorylated Rb accumulation and thus DNA synthesis inhibition. A major inhibitor used to do the same is Nutlin-3. Similar to MDM2, MDMX is often found to be upregulated in retinoblastoma. The induction of MDMX in retinoblastoma cell lines like Y79, Weri1 and ML-1 showed increased expression of p53, phospho-p53 [ser-15], and various p53 targets like p21, and MDM2. Retinal cells lacking Rb and p107 also showed cell proliferation and survival when expressed with MDMX [19].

MDM4 is another protein which along with MDM2 maintains the stability of p53. Mutations and polymorphisms on MDM4 are studied vividly as this protein regulates Rb levels through MDM2 mediated ubiquitination [76]. Further small molecule inhibitors like CEP134 have shown that MDM4 expressing retinoblastoma cell lines depicted tumour regression by activation of p53 pathway [77]. Another molecule that acts as an inhibitor of p53 is HDM2. P53 protein auto-regulates itself through the transcription of these inhibitor proteins. Thus, loss of HDM2 leads to the subsequent accumulation of the p53 and thus the apoptosis of these tumour cells. Strategies that try to enhance the expression of HDM2 are thus often tried in retinoblastomas with wild type p53 [78]. The role of p53 in modulating cell plasticity is well-established. Several studies conferred to p53 a key role in the regulation of EMT mainly through modulating the complex miRNAs network [82–85]. On the other hand, p53 has been proposed to be a critical player in the regulation of stemness, differentiation, reprogramming of pluripotent stem cells, and inhibiting cancer stemness [79]. Interestingly, concomitant inactivating mutations in both p53 and Rb genes results in less differentiated tumours compared to a WT p53 background, suggesting the existence of a functional interrelation [80, 81]. In this regard, MEFs lacking Rb function in a genetic context of p53 KO, showed increased sphere formation in 3-D culture, suggesting that double Rb/p53 inactivation fosters the acquisition of a stem-like phenotype [82]. These studies suggest that genetic interplay between Rb and p53 plays a key role in regulating the undifferentiated state in both normal and tumour cells.

### Wnt signalling

The Wnt signaling is one of the major signaling pathways known to maintain the tissue morphogenesis during embryogenesis, stem-like properties, and DNA repair. In the CNS, Wnt signaling is required for cell fate specification, axon guidance, synapse development and the establishment of neuronal circuits [83]. A number of studies have shown that Wnt signaling pathway controls stem/progenitor cells. In this regard, Wnt3a can regulate the self-renewal of hematopoietic stem cells [84], the adult neurogenesis in the hippocampus [85], and the proliferation of retinal stem cells [86]. In another study it has been reported that canonical β-catenin/Wnt pathway improves retinal pigmented epithelium derivation from human embryonic stem cells [87]. Due to its ubiquitous presence in various cancers, manipulation of this pathway is often considered as an anti-neoplastic strategy in cancer treatment. During the growth of retinal cells, the Rb and Wnt pathway are supposed to act in opposite manner to control their proliferation and differentiation. Studies on developing chick retina found that inhibition of Wnt signaling caused blockage of premature neuronal differentiation and proliferation of progenitor cells. Overexpression of Wnt2b results in neuronal differentiation indicating that the mutations responsible for the deregulation of this pathway in RB1 knockout retinal progenitor cells could lead to tumorigenesis [88]. Studies on murine models showed that deletion of Rb homologs like p107 and p130 causes an increment in β-catenin expression, indicating the crosstalk between Rb and Wnt pathway. It has also been shown that cyclin D1, a downstream molecule of the Wnt signaling is a regulator of Rb pathway [89]. Wnt is negatively controlled by a tumor suppressor MEG3, making it a prognostic factor to detect retinoblastoma progression [90]. The silencing of PRC1 gene in HXO-RB44 and WERI-Rb-1 cell lines showed downregulation of Wnt signaling leading to a lower expression of PRC1, VEGF, Wnt1, β-catenin, cyclinD1, and GSK-3β phosphorylation, thus decreased proliferation and invasion abilities. This study showed the possibilities of suppressing the proliferation, and angiogenesis by downregulating the Wnt/β-catenin pathway in retinoblastoma cells [91]. Similar studies done using siRNAs to repress SOST expression indicated the upregulation of various genes like Wnt-1, β-catenin, c-Myc, cyclinD1, MMP-2 and MMP-9, further indicating the promotion of proliferation, invasion, migration, and inhibition of apoptosis in human retinoblastoma cells by the activation of the pathway [92]. The tumor suppressor role of Apc2 was also observed in retinoblastoma cell lines. Apc2 was found hypermethylated in 70% of tumor samples, and as a result, Wnt is activated in retinoblastoma [93]. A number of studies reported that Wnt signaling pathway confers plasticity to retinal pigment epithelium by controlling EMT and metabolic rewiring. In this regard, a study led by Tseng et al showed that Wnt induces EMT in ARPE-19 cells upon loss of contact inhibition [94]. In another study it has been observed that laser photocoagulation fosters Wnt/β-catenin signal transduction pathway, thus sustaining both retinal pigment epithelium (RPE) proliferation and EMT. Wnt/β-catenin signaling also induces the expression of transcription factors required for RPE biogenesis [95]. Wnt signaling is also linked to Warburg Effect and cancer metabolic rewiring [96]. In this regard, Wnt may foster the expression and the activity of glycolytic enzyme phosphofructokinase 1 platelet isoform (PFKP) in a β‑catenin‑independent manner. In a study by Vallée et al. conducted in exudative age-related macular degeneration, it has been reported that WNT/beta-catenin pathway stimulates PI3K/Akt and HIF-1α signaling which in turn leads to the activation of glycolytic enzymes [97].

### Ras/MEK/ERK pathway

Considered to be the best characterised signalling pathway in cell biology, Ras/Raf pathway transduces signals from the extracellular area to the nucleus and enables activation of cell growth, differentiation, and migration. Studies have shown that Rb protein is a nuclear target of this signalling. The levels of Rb in turn are known to regulate the Ras expression. Rb deficient cell lines expressed elevated levels of Ras during its G1 phase, but this was found to be reversed in the presence of a defective E2F expression [98]. Cyclin dependent kinase regulatory subunit 1B (CKS1B) is a protein whose downregulation efficaciously inhibits the proliferation, invasion and angiogenesis of retinoblastoma cell lines through MEK/ERK signalling pathway. Activation of the MEK/ERK signalling enhances the expression of MEK, ERK, BCl2, PCNA, cyclinD1, VEGF and b-FGF, leading to increment in cell proliferation, migration, invasion and apoptotic inhibition [99]. Inhibition of various kinases in Y79 cell lines like CDK2/6, and cyclinD1 by increasing the expression of their inhibitors p21 and p27 have depicted an enhancement in the phosphorylation of JNK and p38-MAPK, NF-κB leading to activation of both pathways [100]. Similar results were observed when using curcumin as an anti-cancer therapeutic molecule [101]. Studies determining the role of BRAF mutations in human retinoblastoma cell found that, the lack of their presence was in accordance with the hypermethylation status of RASSF1 and the presence of CpG island methylator phenotype (CIMP) [102]. Astrocyte Elevated Gene 1 (AEG1) is another protein which when downregulated using RNA interference showed regression of tumor by inhibiting ERK [102]. Other than these, various miRNAs have also been observed that can regulate RB cell growth and metastasis by suppressing the insulin like growth factor-1 receptor IGF1R/k-Ras/Raf/MEK/ERK signalling pathway.

Although direct evidence does not exist in retinoblastoma disease, it is widely described that MAPK pathway might participate in the regulation of cancer cell plasticity and EMT in other tumors. For example, p38-MAPK family has been reported to foster EMT and increase CSCs population in glioma and breast cancer [103, 104]. Pharmacological inhibition of ERK enhances stemness of NSCLC cells via promoting Slug-mediated EMT [105]. In breast cancer models ERK2 can sustain EMT plasticity through DOCK10-dependent Rac1/FoxO1 activation [106] and TNF-α activates EMT program in oral squamous carcinoma by up-regulating P38 and ERK proteins thus enhancing tumor invasion and migratory capabilities [104].

### Notch pathway

Notch is an evolutionarily conserved signalling pathway that promotes proliferative signalling during neurogenesis. In the developing brain, Notch signalling plays a critical role in preserving neural progenitors in an undifferentiated state, by limiting neurogenesis. Notch signalling also controls synaptic plasticity, learning and memory in the adult brain [107]. In addition, a study by Hitoshi et al. suggested that Notch pathway molecules are essential for the maintenance of neural stem cells [108]. Usually notch receptors inhibit photoreceptor differentiation during retinal development and help in maintaining its progenitor state. Thus, during retinal development Notch1 and Notch3 are expressed in the central portion, while Notch2 is mostly present in the periphery. Deregulation of their expression is often observed in retinoblastoma cells in both humans and murine, making this pathway a favourite therapeutic target. Jagged1 is a protein that gets expressed distinctly at distinct time intervals during retinal cell development. Studies have shown that Notch1 and Jag2 are highly expressed in the SO-Rb50 cell line [109]. The overexpression of Jag2 causes an increase in the expression of Hes1, a downstream molecule of the Notch signalling. The suppression of Jag2 expression was also observed to decrease the PI3KC2β mRNA levels, which further promoted AKT expression [110]. MCL1, another anti-apoptotic molecule is found to be degraded in the presence of small molecule inhibitors that decrease the expression of Spleen Tyrosine Kinase (SYK). The inhibition of Notch pathways in WERI Rb1 and Y79 cell lines detected reduction in the cell viability. The combination of these along with exposure to hypoxia is found to be very effective as a treatment strategy against retinoblastoma [111].

Studies on various non-coding RNAs have also deciphered their role in proliferation, stemness, migration and invasion of retinoblastoma cells via the regulation of Notch signalling [112]. Interestingly, Notch signaling pathway has been found to be a critical player of cellular plasticity through the induction of EMT. Niessen et al., showed that Notch-induced expression of Slug plays an important role in the initiation of EMT [113]. In another study, it has been reported that a complex integration of the hypoxia and Notch signaling pathways are necessary for EMT control [114]. Indeed, Notch drives hypoxia-inducible factor 1α (HIF-1 α) recruitment to the lysyl oxidase (LOX) regulatory sequences, thus fostering the hypoxia-induced up-regulation of LOX and the stabilization of Snail-1 protein. Moreover, it has been described that Notch signaling pathway plays a key role in proliferative vitreoretinopathy (VR) formation by fostering EMT program of RPE cells [115]. It has been reported that Notch signaling is an essential player involved in metabolic flexibility and may lead to glycolytic switch through multiple mechanisms. Indeed, hyperactivated Notch signaling induces glycolysis through the activation of AKT whereas hypo-activated Notch limits mitochondrial function and promotes glycolytic metabolism in a p53-dependent way. Intriguingly, it has been observed that only tumours with hyperactivated Notch signaling display an aggressive phenotype since they maintain the ability to revert to mitochondria metabolism [116].

## Conclusion and future perspective

Retinoblastoma is a rare cancer that attracts physicians and scientists because of the potential for its cure and novelty in its treatment modalities. The care for retinoblastoma has been revolutionised by tumour imaging. The similarities observed between the intraocular microenvironment and brain, makes it a possibility to use the paratopic xenograft, since it is a better option when compared to the existing subcutaneous xenografting technique. This can be made possible by the powerful combination of fundus imaging and optical coherence tomography (OCT), and the other uses and advantages of the same need to be further explored. The use of radio-labelled amino acid tracers for molecular imaging using positron emission tomography (PET) and single photon emission computed tomography (SPECT) have shown good results in brain tumour, indicating the possibilities of this being a probable imaging option for retinoblastoma [117]. Furthermore, imaging studies done on high resolution scale might also reveal other imaging biomarkers leading to proper stratification of retinoblastomas for developing improved prognostication and treatment decisions. This could also be used to analyse and impact identification of MYCN retinoblastoma [32].

The level of knowledge we have of the clinical behaviour of intracranial tumours in retinoblastoma is inordinate when compared to our understanding of their molecular features. The fact that even after being one of the oldest cancers to be identified, there is no proper molecular targeted therapy for the disease points to all the unknown secondary mutations that are part of retinoblastoma which are still to be deciphered. Yet, significant progress has been made in the knowledge of the retinoblastoma biology, leading to the discovery and development of small molecules for the treatment of the same, like inhibitors of the MDMX–p53 response (nutlin-3a), histone deacetylase (HDAC) inhibitors, spleen tyrosine kinase (SYK) inhibitors etc. Thus, therapeutic strategies against retinoblastoma have rapidly evolved in the recent years, with a paradigm shift in standard treatment protocols toward the targeted delivery of chemotherapeutic agents. The use of nanotechnology as a prominent delivery system to transport these molecules to the tumour sites has shown the potential to reduce the problems faced by these standard techniques [118–121]. Yet our understanding on various matters regarding the tumour biology and effective therapies need to progress in order to make sure that retinoblastoma is a curable childhood cancer.

## Data Availability

Not applicable.
